# Two‐pronged reversal of chemotherapy resistance by gold nanorods induced mild photothermal effect

**DOI:** 10.1002/btm2.10670

**Published:** 2024-04-18

**Authors:** Qi Shang, Ziyan Chen, Jing Li, Mingmei Guo, Jiapei Yang, Zhu Jin, Yuanyuan Shen, Shengrong Guo, Feihu Wang

**Affiliations:** ^1^ School of Biomedical Engineering, Shanghai Jiao Tong University Shanghai People's Republic of China; ^2^ School of Pharmacy, Shanghai Jiao Tong University Shanghai People's Republic of China; ^3^ Department of Pharmacy Putuo People's Hospital Shanghai People's Republic of China

**Keywords:** chemotherapy, gold nanorods, multidrug resistance, NIR irradiation, photothermal effect

## Abstract

Chemotherapy treatment outcomes are severely restricted by multidrug resistance (MDR), in which tumors develop a multiple cross‐resistance toward drug involving the pump and nonpump resistance mechanisms, resulting in drug efflux and defending against drug toxicity. Herein, we constructed a pH and near infrared (NIR) light responsive nanomedicine DOX@FG based on gold nanorods (GNRs) that demonstrated the potential to improve chemotherapy outcomes by overcoming MDR. DOX@FG was constructed by conjugating folic acid (FA) and doxorubicin (DOX) derivatives onto GNRs, where the DOX derivatives possessed an acid‐labile hydrazone bond. Stimulated by the acidic media in endocytic organelles, DOX@FG exhibited a responsive dissociation for the controlled release of chemotherapeutic DOX. Surprisingly, we found the mild photothermal effect elicited by GNRs under NIR irradiation simultaneously inhibited the pump and nonpump resistance mechanisms, enhancing the intracellular DOX accumulation and sensitizing the cancer cells to DOX, collectively amplify the chemotherapy efficacy and delay the MCF‐7/ADR breast tumor growth. This intelligent DOX@FG nanomedicine with the potential for two‐pronged reversal of MDR may provide a prospective way to encourage chemotherapy efficacy.

**Translational Impact Statement**

Chemotherapy treatment outcomes are severely hampered by intrinsic and acquired MDR. In this study, we show that DOX@FG in the presence of NIR irradiation reversed the MDR from the pump and nonpump resistance mechanisms, enhancing the cancer cell sensitivity to DOX and inhibiting the MCF‐7/ADR breast tumor growth. The results support the potential of the approach in overcoming MDR to amplify the efficacy of chemotherapy.

## 1 INTRODUCTION

Chemotherapy is a leading strategy for cancer treatment in clinical practice.^1^, ^2^, ^3^, ^4^, ^5^, ^6^ However, treatment outcomes are severely hampered by intrinsic and acquired MDR, where tumors develop resistance to a variety of antineoplastic drugs due to a heterogeneous population of drug‐sensitive and drug‐resistant cells.^7^, ^8^ MDR is regulated by pump and nonpump resistance mechanisms, resulting in drug efflux and defending against drug toxicity, respectively.^9^, ^10^ Drug efflux is caused by adenosine triphosphate (ATP)‐binding cassette drug transporters especially P‐glycoprotein (P‐gp) that are overexpressed on the cancer cell membrane,^11^, ^12^, ^13^ which transports the chemotherapeutics to the extracellular matrix, causing the drug concentration to decrease below its therapeutic threshold and attenuate the cytotoxic drug response on tumor cells.^10^, ^14^, ^15^ Although inhibition of drug efflux pumps can increase intracellular drug concentration, MDR can still occur through the activation of cellular antiapoptotic signaling with nonpump resistance mechanisms mainly through the upregulation of B cell lymphoma‐2 (Bcl‐2) and thus restricted the activation of caspase‐7, which acts as an effector enzyme of conserved cysteine proteases (caspases) and a major executioner in the apoptotic process, thereby preventing cancer cells from apoptotic responses to chemotherapy.^16^, ^17^ Accordingly, strategies such as the combination of P‐gp or Bcl‐2 inhibitors with chemotherapy drugs is commonly used to overcome MDR.^9^, ^10^ However, suppression of P‐gp or Bcl‐2 signal alone by conventional medicinal agents has demonstrated poor clinical outcomes because both pump and nonpump resistance can occur independently within a tumor microenvironment.^18^, ^19^, ^20^ Therefore, it is expected that simultaneous P‐gp and Bcl‐2 signaling blockade could improve therapeutic efficiency through suppressing multiple mechanisms of MDR.

Previous investigations have demonstrated that functional nanoparticles bearing photosensitizers for photothermal conversion could affect intracellular MDR‐related proteins and relieve drug resistance in related cancer cells.^21^, ^22^, ^23^, ^24^, ^25^, ^26^ Chen et al. reported that hollow carbon nanospheres with mild photothermal stimuli under near infrared (NIR) irradiation catalyzed free radicals to generate heat shock factor‐1 protein homotrimers, which limited the function of P‐gp and mutant p53 proteins.^27^ Additionally, Li et al. constructed the light‐triggered micelles containing a cisplatin prodrug and cypate to inhibit the expression of MDR‐associated protein 1, which alleviated drug efflux for enhanced ablation of cisplatin‐resistant tumor cells.^28^ In particular, gold nanorods (GNR) is a promising platform for light‐based therapies because of their large surface‐to‐volume ratio and high absorption efficiency in the NIR region,^29^, ^30^, ^31^, ^32^, ^33^, ^34^, ^35^ and can mediate the production of reactive oxygen species or hyperthermia effects upon receiving controlled NIR photons irradiation to elicit antitumor efficacy, effectively inhibiting tumor growth.^36^, ^37^ In contrast to directly damaging tumors with hyperthermia generated by GNRs, we hypothesize that GNRs can regulate the MDR effect under mild photothermal stimulation to enhance intracellular chemotherapeutic agent accumulation and cytotoxicity.

To further optimize chemotherapy outcomes, drug delivery systems provide a prospective strategy for enhancing drug stability and controlling the pharmacokinetic properties of therapeutic agents.^38^, ^39^, ^40^, ^41^, ^42^, ^43^, ^44^, ^45^, ^46^ GNRs have been utilized in localized noninvasiveness treatment as well as drug delivery due to their unique optical property and tunable surface chemistry.^47^, ^48^, ^49^, ^50^, ^51^ Of note, GNRs serve as promising delivery vehicles with good biocompatibility, high loading efficacy, and facile functionalization, that enable delivery of small molecule drugs and biomacromolecular agents to desired target tissues.^52^, ^53^, ^54^, ^55^ Specifically, Au‐thiol bonding interaction is commonly utilized in surface functionalization with drugs or polymers to control the drug release kinetics in GNRs.^56^, ^57^, ^58^ Additionally, GNRs modified with targeted molecules, such as folic acid (FA) and hyaluronic acid, are capable of enhancing drug targeting selectively toward tumor cells and reducing adverse systemic side effects on healthy tissues.^59^, ^60^, ^61^, ^62^, ^63^

**FIGURE 1:**
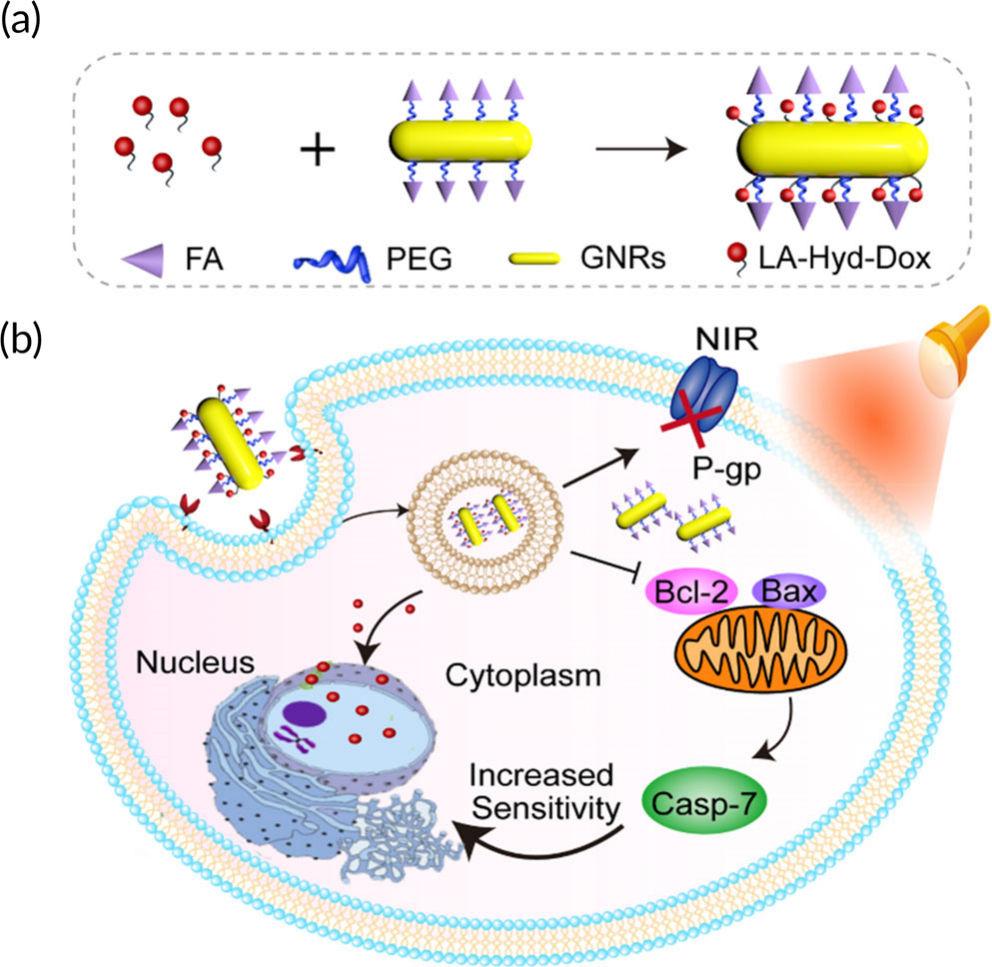
Schematic illustration of DOX@FG reversed chemotherapy resistance via mild photothermal stimuli under near infrared (NIR) irradiation. (a) Preparation illustration of DOX@FG that folic acid (FA)‐PEG‐SH and LA‐Hyd‐DOX derivatives were anchored onto gold nanorods (GNRs) through Au‐thiol bond, in which doxorubicin (DOX) was conjugated onto lipoic acid (LA) via a hydrazone linker. (b) The local mild photothermal effect initiated DOX@FG to combat multidrug resistance (MDR) by simultaneously downregulating the expression of P‐gp and Bcl‐2, further enhancing the intracellular DOX accumulation and improving the sensitivity of cancer cells to DOX.

In this study, we proposed a pH and NIR‐responsive nanomedicine delivery system DOX@FG combating MDR to improve chemotherapeutic outcomes. DOX@FG was constructed by anchoring FA‐PEG‐SH and LA‐Hyd‐DOX derivatives onto GNRs through Au‐thiol interactions (Figure 1a), in which DOX was conjugated to α‐lipoic acid (LA) via an acidic environment‐sensitive hydrazone linker, which was stable under alkaline and neutral conditions, while hydrolyzed in an acidic medium of endosomes or lysosomes to form the corresponding ketones and hydrazides, thereby favoring controlled drug release. Importantly, we found the mild photothermal effect generated by GNRs simultaneously downregulated the levels of P‐gp and Bcl‐2 under NIR (Figure 1b). As demonstrated in the MCF‐7/ADR cell model, DOX@FG under NIR effectively reduced DOX efflux in tumor cells to promote intracellular DOX accumulation and retention, as well as enhanced the sensitivity of cancer cells to DOX. Therefore, DOX@FG initiated a photothermal effect under laser irradiation that efficiently reversed MDR in terms of pump and nonpump mechanisms, while amplifying the chemotherapeutic efficacy on MCF‐7/ADR cells.

## 2 MATERIALS AND METHODS

### 2.1 Materials and animals

Doxorubicin hydrochloride (DOX·HCl, 98%), hydrazine hydrate, LA, FA (98%), acetyl chloride, and ethylenediamine were obtained from Aladdin Industrial Inc. HS‐PEG‐COOH (Mw = 3500) was purchased from Beijing JenKem Technology Inc. Hoechst 33342, fetal bovine serum, penicillin–streptomycin, RPMI 1640 medium, and trypsin–EDTA solution (0.25%) were obtained from Beijing Solarbio Science and Technology Co., Ltd. The female BALB/c nude mice (18–22 g) were purchased from the laboratory animal center of Fudan University. Animal experiments were carried out following the animal protocol approved by the animal care and welfare committee of Shanghai Jiao Tong University (SYXK (Hu) 2018–0028).

### 2.2 Synthesis of FA‐modified GNRs (FG)

The FG was prepared by conjugating FA‐tethered thiol polyethylene glycol (HS‐PEG‐FA) derivatives onto GNRs via Au‐thiol interaction. The GNRs were prepared using a seed‐mediated growth method following the previous investigations.^13^, ^57^, ^64^ Then, 11‐mercaptoundecanoic acid (MUDA) was added into the GNRs‐CTAB solution to obtain GNRs‐MUDA by ligand exchange. To prepare FA derivatives, the 30 μmol HS‐PEG‐COOH (105 mg) reacted with 3 mmol ethylenediamine (200 μL) under N_2_ protection to obtain purified HS‐PEG‐NH_2_ after dialysis with H_2_O, which subsequently conjugated with 60 μmol FA (26.5 mg) by an amide reaction. Finally, HS‐PEG‐FA (20 mg) was added into the GNRs‐MUDA solution under N_2_ protection and stirred at room temperature for 24 h; and the FG product was collected through repeated centrifuging. The PG as control group was prepared by conjugating HS‐PEG with GNRs‐MUDA. ^1^H NMR spectra were obtained to confirm the structure of the compounds.

### 2.3 Synthesis of LA‐Hyd‐DOX

The preparation of LA‐Hyd‐DOX was based on our previous research.^65^ Briefly, 2 mmol LA reacted with acetyl chloride (0.35 mL) in anhydrous methanol to obtain a lipoic acid ester. This molecule then reacted with hydrazine hydrate (85%, 10 mL) in anhydrous ethanol and purified through reduced pressure distillation to obtain lipoic acid hydrazide (LA‐Hyd). Finally, 1 mmol LA‐Hyd added into 1 mmol DOX in dimethyl sulfoxide (DMSO) solution and stirred for 24 h; LA‐Hyd‐DOX was then purified by precipitation of the product with acetonitrile.

### 2.4 Preparation and characterization of DOX@FG

The LA‐Hyd‐DOX solution reacted with the FG and PG compounds to prepare DOX@FG and DOX@PG by Au‐thiol bonds, respectively. The mixture of 8 μM LA‐Hyd‐DOX (500 μL) and 500 pM FG (500 μL) was stirred for 24 h and then centrifuged at 15,000 rpm for 15 min to remove free LA‐Hyd‐DOX to obtain DOX@FG. Transmission electron microscopy (TEM) was used to characterize the shape and size particle of DOX@FG. The absorbance spectra of GNRs, FG, DOX, and DOX@FG were detected by UV–Vis; and the fluorescent intensity change of LA‐Hyd‐DOX (8 μM), after reacting with FG at concentrations from 0 to 550 pM, was evaluated at the excitation wavelength of 480 nm. To assess the stability, DOX@FG was incubated in phosphate buffered saline (PBS) with 10% fetal bovine serum (FBS) at 37°C. At predetermined time points, the particle size of DOX@FG was measured by Zetasizer (Nano ZS, Malvern).

### 2.5 Cumulative DOX release

To simulate the DOX responsive release profile, DOX@FG was suspended in 5 mL buffer medium at pH 7.4, 6.8, and 5.0, which represented the microenvironment of normal cells, tumor tissue, and tumor cells, respectively. In all three experimental conditions, DOX@FG was placed in dialysis tubing (molecular weight cut‐off = 3.5 kDa), immersed in 45 mL buffer medium, and then stirred (37°C, 100 rpm). At predetermined time points, 1 mL release media was obtained to detect the cumulative release amount of DOX from DOX@FG through high performance liquid chromatography (HPLC).

### 2.6 Cell culture

MCF‐7 and MCF‐7/ADR cells were cultured in RPMI 1640 medium with 10% FBS and 1% penicillin–streptomycin and maintained at 5% CO_2_ at 37°C. When the cells grew to high density (80%), they were digested with 0.25% (w/v) trypsin digestion, passaged at a ratio of 1:3 with culture medium.

### 2.7 Intracellular DOX accumulation

To analyze the cellular uptake ability of the nanomedicine system, MCF‐7/ADR cells (2 × 10^5^) were incubated in 12‐well plate overnight. After replacing fresh medium, cells were incubated with DOX@PG, DOX@FG, and 1 mM free FA + DOX@FG at DOX concentration 2.5 μg/mL for 2 h. The mean number of GNRs per cell was quantified by inductively coupled plasma mass spectrometry (ICP‐MS). Additionally, after incubating MCF‐7/ADR cells with DOX@FG for 3 h, the cellular release of DOX (red fluorescence) was imaged via confocal laser scanning microscopy (CLSM). The acidic organelles were stained with lysotracker green (green fluorescence), and the cell nuclei were counterstained with Hoechst 33342 (blue fluorescence). Intracellular DOX fluorescence intensity in MCF‐7/ADR cells after incubation with free DOX (2.5 μg/mL), DOX@PG, and DOX@FG for various periods was quantified by flow cytometry.

### 2.8 Cytotoxicity assay

The cell inhibition rate of DOX@FG, DOX@PG, and free DOX at different concentrations was evaluated by using MCF‐7 and MCF‐7/ADR cells (5 × 10^3^) in a 96‐well plate. After 48 h incubation, 15 μL of 5 mg/mL MTT was added to each well. The cells were incubated for an additional 4 h and then the medium was discarded. Thereafter, 200 μL of DMSO was added to each well to dissolve the formazan crystals under stirring for 10 min. The absorbance at 570 nm was detected with a microplate reader and cell viability and IC50 values were calculated.

### 2.9 Temperature curve

The temperature change was evaluated at a series of power densities (1.0 W/cm^2^, 2.0 W/cm^2^, 3.0 W/cm^2^). First, the FG (20 μg/mL GNRs) and water were incubated in 48‐well plate for corresponding timepoints (30, 60, 90, 120, 150, and 180 s); then the nanomedicine system was irradiated by a 808 nm NIR laser. The temperature of the solution was measured by a digital thermometer, and the temperature elevation curve was analyzed.

### 2.10 Cell viability under NIR

The MCF‐7/ADR cell viability after undergoing NIR irradiation was determined by the MTT assay. After exposing the cells to FG (20 μg/mL GNRs) for 6 h, MCF‐7/ADR cells were irradiated by a 808 nm NIR laser at a series of power densities (1.0, 2.0, 3.0, and 4.0 W/cm^2^) for 180 s. Afterward, MTT was used to incubate with cells for 4 h and then 200 μL DMSO was added into cells without medium to dissolve formazan crystals under stirring for 10 min. The absorbance at 570 nm was detected with a microplate reader to determine cell viability ratio. Additionally, changes in the viability of MCF‐7/ADR cells after being treated with FG (20 μg/mL GNRs), DOX (2.5 μg/mL), DOX@PG, and DOX@FG in the presence or absence of NIR laser irradiation (2.0 W/cm^2^, 180 s) was detected by a microplate reader.

### 2.11 Cellular uptake under NIR

MCF‐7/ADR cells were treated with DOX or DOX@FG (20 μg/mL GNRs, 2.5 μg/mL DOX) for 6 h and then irradiated by a 2.0 W/cm^2^ laser for 180 s. Control experimental groups consisted of MCF‐7/ADR cells treated with identical concentrations of DOX or DOX@FG, without subsequent NIR irradiation. The cellular uptake of DOX and DOX@FG in MCF‐7/ADR cells were imaged by CLSM and analyzed with a fluorescence signal (blue: Hoechst 33342 stained nuclei; red: DOX).

### 2.12 Cellular DOX accumulation and retention under NIR

To study the effect of NIR irradiation on the accumulation of DOX in MCF‐7/ADR cells, cells were incubated with DOX (2.5 μg/mL), DOX + FG (20 μg/mL GNRs), or DOX@FG for 6 h. Then, cell suspension was collected and treated with NIR irradiation at 2 W/cm^2^ for 180 s. Afterwards, cells were continuously incubated with drug containing culture medium to investigate the cellular DOX accumulation. To investigate the intracellular DOX retention behavior, MCF‐7/ADR cells were pretreated with DOX, DOX + FG, or DOX@FG, and then incubated with drug free culture medium after NIR irradiation. To analyze intracellular DOX content, cells were digested and dispersed in acetonitrile/water solution (1:1, v/v), and collected the supernatant after centrifuging at 10,000 rpm for 5 min. The accumulation or retention of DOX in MCF‐7/ADR cells were then analyzed by HPLC.

### 2.13 Western blot analysis

To investigate the sensitivity of MCF‐7/ADR cells incubated in the presence of FG, cells were received different treatments of DOX, DOX + FG, and DOX@FG (20 μg/mL GNRs, 2.5 μg/mL DOX) in the presence or absence of NIR laser irradiation; and the expression of P‐gp, Bcl‐2, and activated caspase‐7 in MCF‐7/ADR cells was analyzed by western blotting.

### 2.14 Apoptosis detection

MCF‐7/ADR cells (1 × 10^5^) were cultured overnight in 12‐well plates and treated with FG, DOX, and DOX@FG (20 μg/mL GNRs, 2.5 μg/mL DOX) for 6 h. Afterwards, cells were treated with laser irradiation (2.0 W/cm^2^, 180 s); whereas the control group was not treated with the laser irradiation. After 48 h, cells were treated with 4 μL Annexin‐V FITC (10 mg/mL) and 100 μL PI (0.5 mg/mL) for 30 min in the dark and the apoptosis of MCF‐7/ADR cells upon these different treatments were detected by flow cytometry.

### 2.15 In vivo biodistribution

To detect the biodistribution of DOX@FG in vivo, MCF‐7/ADR tumor‐bearing BALB/c nude mice intravenously received DOX@FG (2.5 mg/kg DOX) and their blood vessels were imaged with photoacoustic (PA) tomography at predetermined timepoints (1.5, 3, 6, and 12 h). After intravenous injection of DOX@PG and DOX@FG at 6 h, the distribution of the nanoparticles in the mice's major organs and tumor tissues were determined by ICP‐MS and quantified based on the amount of Au in corresponding tissues or organs.

### 2.16 Antitumor effect in vivo

To investigate the therapeutic efficacy, female BALB/c nude mice were used to construct a DOX‐resistant MCF‐7/ADR tumor model. The female BALB/c nude mice (18–22 g) were subcutaneously inoculated with MCF‐7/ADR cells in the right axilla, and then the mice were randomly divided into five groups (*n* = 6) when the tumor grew to around 100 mm^3^. After grouping, the mice were injected with saline, DOX, FG + NIR (2.0 W/cm^2^, 3 min), DOX@FG, or DOX@FG + NIR on days 0, 3, 6 and 9, where the DOX dose was standardized to 2.5 mg/kg in the DOX, DOX@PG, and DOX@FG groups, and the GNRs dose was standardized to 20 mg/kg in the FG and DOX@FG groups. Corresponding infrared thermal images was carried out on mice treated with saline, FG + NIR, and DOX@FG + NIR. The tumor size was measured every 4 days and the tumor volume was calculated by the formula: *V* = (major axis) × (minor axis)^2^/2. Mice were euthanized when tumor volume reached 2 cm^3^ or when body weight loss exceeded more than 20%. Finally, tumors extracted from the mice were obtained and tumor weights were weighed and western blotting was used to analyze the P‐gp and Bcl‐2 levels under various groups at the end of the experiments. In addition, hematoxylin and eosin (H&E) and terminal deoxynucleotidyl transferase dUTP nick end labeling (TUNEL) staining were used to detect necrosis and apoptosis of tumor cells.

### 2.17 Statistical analysis

Data were presented as mean ± SD. Statistical analysis was performed using GraphPad Prism software 8. The two‐tailed unpaired *t*‐test was used to determine statistical significance between two treatment groups and one‐way or a two‐way ANOVA was used for multiple comparisons. *p < 0.05 and **p < 0.001.

## 3 RESULTS AND DISCUSSION

### 3.1 Preparation and characterization of DOX@FG

**FIGURE 2:**
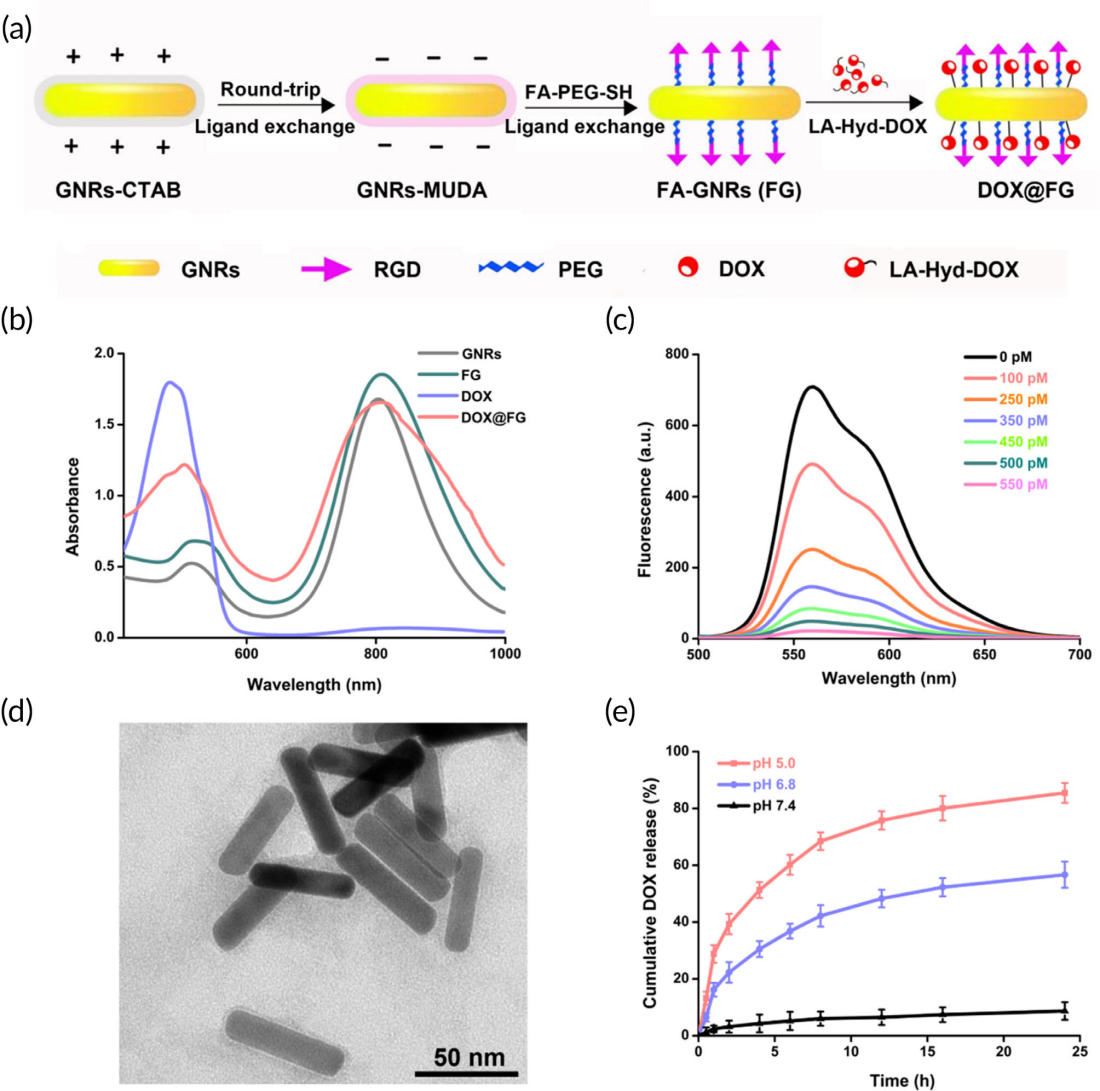
Preparation and characterization of DOX@FG. (a) Schematic illustration of the fabrication of DOX@FG. (b) UV–vis absorbance spectra of gold nanorods (GNRs), FA‐modified GNRs (FG), doxorubicin (DOX), and DOX@FG. (c) Change in fluorescent intensity of α‐lipoic acid (LA)‐Hyd‐DOX (8 μM) after reaction with FG at different concentrations from 0 to 550 pM, excitation wavelength = 480 nm. (d) Transmission electron microscopy (TEM) image of DOX@FG demonstrating one‐dimensional nanorods. (e) In vitro release profile of DOX from DOX@FG at 37°C (*n* = 3).

The DOX@FG nanomedicine was constructed by anchoring targeting ligand FA derivatives (FA‐PEG‐SH) and pH‐responsive chemotherapeutic drug DOX derivatives (LA‐Hyd‐DOX) onto GNRs via Au‐thiol interactions (Figure 2a). Briefly, GNRs were prepared by the CTAB templating strategy; and subsequently MUDA was added into the GNRs‐CTAB solution to obtain GNRs‐MUDA by ligand exchange. To conjugate the FA onto GNRs, FA was reacted with PEG via amine bonds to obtain FA‐PEG‐SH, according to the scheme illustration in Figure S1; and then FA‐PEG‐SH solution was mixed with GNRs‐MUDA to obtain FA‐modified GNRs (FG) by ligand exchange (Figure S2). ^1^H NMR confirmed the correct structure of FA‐PEG‐SH (Figures S3 and S4). LA‐Hyd‐DOX, with an acid‐labile hydrazone linkage was conjugated onto FG by Au‐thiol interactions to obtain DOX@FG with a DOX loading efficiency of 11.1%. As depicted in Figure 2b, UV–Vis spectroscopy demonstrated the overlapping absorption spectra of DOX@FG and DOX, indicating that the intact structure of DOX@FG was retained after chemical modification. Additionally, GNRs could serve as an indicator of fluorescence donors owing to the unique property of the nanosurface energy transfer (NSET) effect.^66^, ^67^ As demonstrated in Figure 2c, the fluorescence intensity of LA‐Hyd‐DOX significantly decreased after reacting with FG at different concentrations, and the signal was almost completely quenched at 550 pM, due to the optical properties of GNRs. The TEM image in Figure 2d showed the regular rod shape of DOX@FG with the surface layer of 2–3 nm thick for polymer modification, the average length and diameter were about 53 and 13 nm, respectively. The aspect ratio of the DOX@FG was calculated to be 4.1. These characterization results of UV–Vis absorption spectroscopy, change in fluorescence intensity, and TEM imaging confirmed the successful synthesis of DOX@FG, suitable for delivery of DOX.

Research has shown that the pH value in endosomes and lysosome is 5.0–6.0 and 4.5–5.0, respectively,^68^, ^69^, ^70^ and thus the introduction of acid‐labile hydrazone linker in DOX@FG facilitated the achievement of controlled release of DOX in intracellular acidic media. To simulate the DOX release profile under physiological condition and acidic media in endocytic organelles, the cumulative DOX release from DOX@FG at 37°C was detected by HPLC at pH 7.4, 6.8, and 5.0, respectively. As shown in Figure 2e, the release amount of DOX from DOX@FG was dependent upon the pH of the buffer medium. The release of DOX@FG at pH 7.4 was slow, with only 8.7% released over 24 h. Apparently, DOX@FG released about 85.5% of loaded DOX after 24 h at pH 5.0 buffer medium, demonstrating promoted DOX release from DOX@FG at low pH, which sensitized the hydrazone bond. The pH‐responsive DOX@FG facilitated targeted drug delivery because it was stable in the blood circulation while responsively releasing free DOX from intracellular acidic media, contributing to improving the accumulation of intracellular DOX. In addition, the particle size of DOX@FG in PBS with 10% FBS showed no obvious changes during the investigated period (Figure S5), indicating the good stability of DOX@FG in blood circulation.

### 3.2 Targeting ability and cytotoxicity of DOX@FG

**FIGURE 3:**
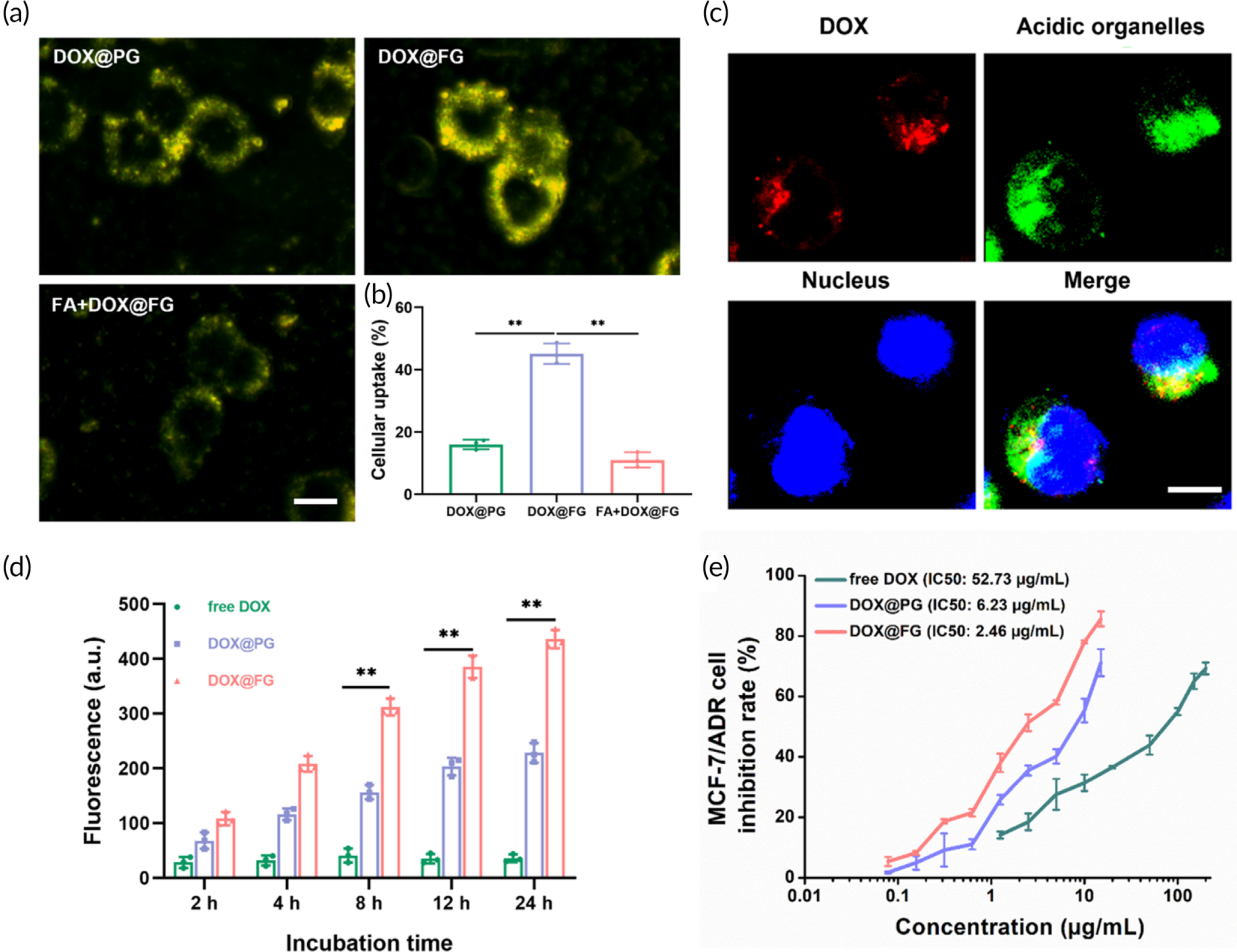
Intracellular doxorubicin (DOX) evaluation under corresponding treatment groups. (a) Dark‐field images of MCF‐7/ADR cells incubated with DOX@PG, DOX@FG, and 1 mM free FA + DOX@FG. Scale bar: 50 μm. (b) Quantification of the gold nanorods (GNRs) uptake rates determined by inductively coupled plasma mass spectrometry (ICP‐MS). (c) Confocal images of cellular DOX release after incubation of MCF‐7/ADR cells with DOX@FG. Scale bar: 10 μm. (d) Flow cytometry results for the mean fluorescence intensity of MCF‐7/ADR cells under various timepoints (*n* = 3). (e) The cell inhibition rate of MCF‐7/ADR cells following treatment of DOX, DOX@PG, and DOX@FG (*n* = 3). **p < 0.001.

Targeted therapy is a pivotal strategy to concentrate the drug in cancer cells through ligand‐receptor interactions, in which the folate receptor (FR) is one of the most prominently epithelial cancer markers.^62^, ^71^, ^72^, ^73^ With the introduction of FA, DOX@FG constituted a targeted intracellular delivery of DOX to breast cancer cells, which overexpress FR on their cell membrane with a high affinity for FA.^74^ To validate the targeting ability of DOX@FG toward MCF‐7/ADR cells, the cellular uptake behavior was investigated after cells were treated with DOX@PG, DOX@FG, and free FA + DOX@FG. As shown in Figure 3a, MCF‐7/ADR cells incubated with DOX@FG displayed much stronger golden color than that of cells treated with DOX@PG, which suggests that FA‐modified DOX@FG can target MCF‐7/ADR cells. The specificity of FA ligand in DOX@FG uptake was further evaluated by treating MCF‐7/ADR cells with the DOX@FG in the presence of free FA. Dark‐field imaging showed that the cellular uptake of FA‐modified DOX@FG was significantly reduced, suggesting that free FA can inhibit the binding of FA‐modified DOX@FG with FR over‐expressed tumor cells, leading to lower cellular uptake. In addition, the quantitative results of the GNRs uptake rates also demonstrated the superior targeting of DOX@FG to MCF‐7/ADR cells (Figure 3b). Collectively, these results indicated that enhanced cellular uptake of FA‐modified DOX@FG could be achieved based on the FR‐mediated endocytosis, and hence the DOX@FG can be used as efficient, targeted intracellular delivery of DOX.

Due to the NSET effect, FG and DOX in DOX@FG could function as the “quencher” and the “donor” respectively, in which the quenched fluorescence of DOX is recovered after the degradation of DOX@FG. Therefore, the fluorescence signal of DOX can serve as an indicator for monitoring the release of the DOX from DOX@FG. As displayed in the confocal images on Figure 3c, the red DOX signal was localized in acidic organelles after incubation of DOX@FG in MCF‐7/ADR cells, which confirmed the sensitive release of DOX from DOX@FG and recovery of the DOX fluorescence signal. To semi‐quantitatively analyze the DOX release, flow cytometry was used to determine the fluorescence intensity of intracellular DOX (Figure S6). Compared to free DOX and DOX@PG, DOX@FG displayed higher DOX fluorescence intensities in both MCF‐7 cells (Figure S7) and MCF‐7/ADR cells (Figure 3d) at all incubation timepoints analyzed. From Figure 3d, the DOX signal in MCF‐7/ADR cells incubated with DOX@FG was 12.11 and 1.91‐fold greater than free DOX and DOX@PG groups at 24 h, respectively. With increased DOX release, the cell viability was significantly inhibited in both DOX@FG treated MCF‐7 and MCF‐7/ADR cells. The greater inhibition rate of MCF‐7 cells exposed to DOX@FG was demonstrated in Figure S8. For MCF‐7/ADR cells, the IC50 was 2.46 μg/mL in the DOX@PG experimental group, which was less than DOX and DOX@PG IC50 values (52.73 and 6.23 μg/mL respectively) in Figure 3e. Intracellular DOX evaluation and cytotoxicity results verified that acid‐sensitive DOX@FG disassociated as DOX in intracellular acidic organelles, causing a greater cytotoxic effect for both MCF‐7 and MCF‐7/ADR cells.

### 3.3 Cell viability after DOX@FG treatment under NIR

**FIGURE 4:**
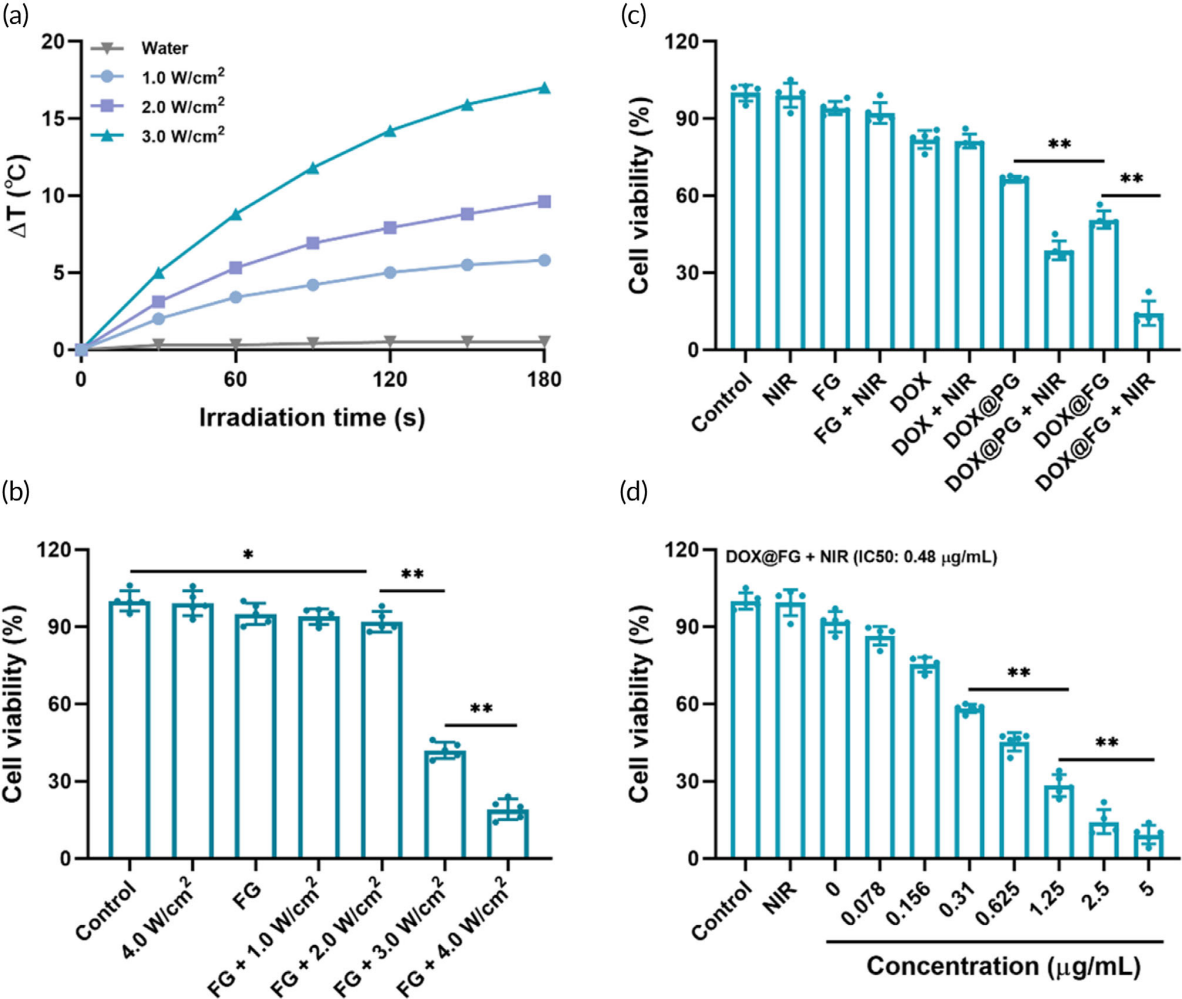
Change in cell viability in the presence of near infrared (NIR) irradiation. (a) Temperature elevation of FA‐modified GNRs (FG) (20 μg/mL gold nanorods (GNRs)) and water irradiated by a 808 nm NIR laser. (b) Cell viability after MCF‐7/ADR cells were treated with FG for 6 h and exposed to 808 nm NIR laser at various intensities (*n* = 5). (c) Changes in MCF‐7/ADR cell viability after treatment with FG, doxorubicin (DOX), DOX@PG, and DOX@FG in the presence or absence of NIR (*n* = 5). (d) Sensitivity of DOX@FG‐treated MCF‐7/ADR cells to DOX after 48 h under laser irradiation (*n* = 5). *p < 0.05 and **p < 0.001.

To evaluate the photothermal effect of DOX@FG in vitro, the temperature elevation profiles were measured at a series of NIR irradiation densities. As illustrated in Figure 4a, the photothermal effect of FG was positively correlated with NIR exposure time and density. After irradiating FG nanoparticles with a 808 nm NIR laser with a density of 3.0 W/cm^2^ for 180 s, the temperature increased to 17°C, which was significantly higher than water; and this confirmed that FG nanoparticles could convert NIR light energy into heat effectively. From Figure 4b, the cell viability significantly decreased with a NIR laser density >3.0 W/cm^2^, demonstrating the effect of laser‐thermal conversion efficiencies on cell viability. To investigate the effect of mild photothermal therapy on MCF‐7/ADR cells, we evaluated the DOX@FG efficacy under a NIR laser at 2.0 W/cm^2^ (180 s) to avoid cells death caused by hyperthermia. As displayed in Figure 4c, the cell viability was slightly inhibited after MCF‐7/ADR cells were treated with FG in the presence of NIR. Conversely, the cell viability in DOX@FG + NIR group decreased significantly to 14.2%, compared to FG + NIR (92%) and DOX@FG (50.6%), indicating DOX@FG increased cytotoxic effects after integrating the efficacy of DOX and FG under NIR irradiation. Furthermore, the sensitivity of MCF‐7/ADR cells to DOX from DOX@FG‐induced mild photothermal effects was investigated. After incubated with MCF‐7/ADR cells for 48 h, the IC50 was 0.48 μg/mL in DOX@FG + NIR experimental group (Figure 4d), which was significantly less than the DOX@FG group (Figure 3d). These results indicated the MCF‐7/ADR cells treated with DOX@FG + NIR developed an increased sensitivity and cytotoxicity to DOX, which may be attributed from the mild photothermal effect induced by DOX@FG in MCF‐7/ADR cells.

### 3.4 MDR‐associated protein expression

**FIGURE 5:**
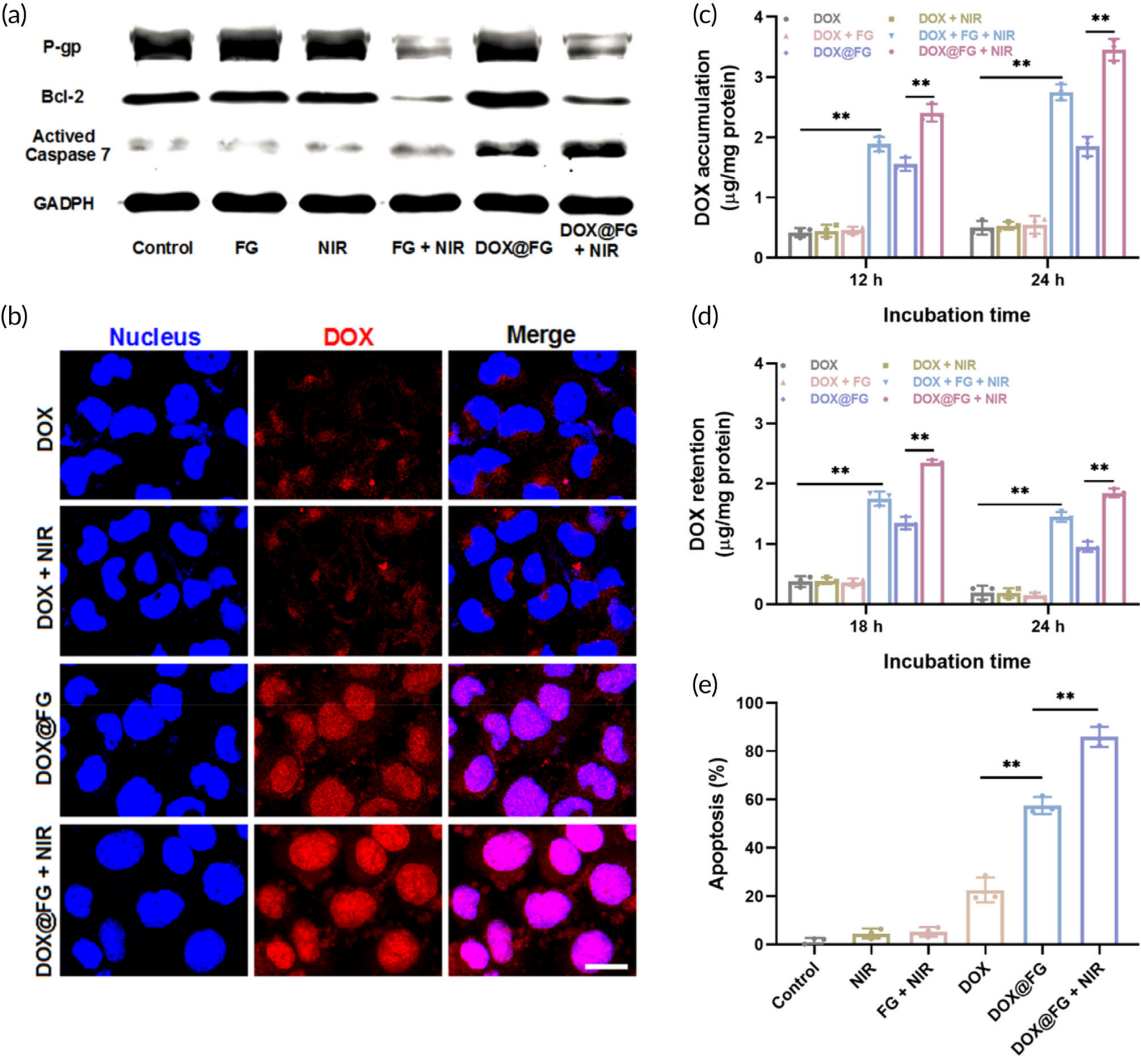
The antitumor efficacy evaluation in vitro. (a) Western blotting analysis of the P‐glycoprotein (P‐gp), B cell lymphoma‐2 (Bcl‐2), and Caspase‐7 levels in MCF‐7/ADR cells upon different treatments. (b) Confocal images of MCF‐7/ADR cells treated with doxorubicin (DOX) and DOX@FG (20 μg/mL gold nanorods (GNRs), 2.5 μg/mL DOX) with or without near infrared (NIR). Scale bar: 20 μm. (c) The effect of laser irradiation on the accumulation of DOX in MCF‐7/ADR cells in the indicated treatments (*n* = 3). (d) Retention of DOX in MCF‐7/ADR cells after preincubation with DOX, DOX + FA‐modified GNRs (FG), or DOX@FG with NIR or not (*n* = 3). (e) Apoptosis of MCF‐7/ADR cells after treated with NIR, FG + NIR, DOX, DOX@FG, and DOX@FG + NIR (*n* = 3). **p < 0.001.

MDR is a leading obstacle limiting the chemotherapy efficacy, which is associated with the upregulation of drug transporters such as P‐gp to efflux drug and the mutation of pro‐apoptosis genes such as P53 to influence the activity of apoptosis regulator Bcl‐2.^75^ In addition, the nuclear factor kappa‐B (NF‐κB) signaling plays a crucial role in triggering chemotherapy resistance by activating the MDR‐associated genes. Previous investigations have demonstrated that the mild photothermal could induce the upregulation of heat shock factor (HSF‐1) trimers and translocate to the cell nucleus to compete with NF‐κB for binding of MDR‐related genes, resulting in the reduction of P‐gp expression and inducing the degradation of mutant P53 and Bcl‐2.^27^, ^53^ To investigate the chemotherapeutic sensitivity under GNR induced mild photothermal effect, MCF‐7/ADR cells were treated with different treatments; and western blotting was used to determine the level of P‐gp and Bcl‐2 in MCF‐7/ADR cells.^76^ As shown in Figures 5a and S9, the expression of P‐gp in MCF‐7/ADR cells decreased when incubated with FG and DOX@FG in the presence of NIR, indicating that the mild photothermal effect induced by FG contributed to reducing DOX efflux to reverse pump resistance mechanisms. However, apoptosis is a dynamic process related to the Bcl‐2 and Bcl‐2 associated X proteins where cancer cells can independently defend against DOX cytotoxicity by enhancing antiapoptotic Bcl‐2 levels. Notably, the Bcl‐2 expression was downregulated while activated caspase‐7 was upregulated in the DOX@FG + NIR group, indicating DOX@FG under NIR irradiation also inhibited nonpump MDR mechanisms. Therefore, DOX@FG demonstrated a two‐pronged reversal of MDR in terms of pump and nonpump mechanisms by simultaneously blocking the P‐gp and Bcl‐2 signaling, demonstrating a promising potential to enhance intracellular DOX accumulation and increase the sensitivity of cancer cells to chemotherapeutics.

### 3.5 Intracellular DOX evaluation under NIR

The cellular uptake of DOX and DOX@FG under NIR was imaged in Figure 5b. Compared to free DOX, MCF‐7/ADR cells demonstrated an increase in fluorescence signal after treatment with DOX@FG, which was attributed to the FA‐mediated tumor‐targeting effect that enhanced the intracellular uptake of DOX@FG. Apparently, the cellular uptake of DOX@FG was increased in the presence of NIR, whereas no effect was demonstrated in the DOX + NIR group, indicating that DOX@FG‐induced a mild photothermal effect through reducing the drug efflux. As demonstrated in Figure 5c, the accumulation of DOX in MCF‐7/ADR cells treated with DOX@FG + NIR was 6.76‐ and 1.86‐fold higher than that in DOX and DOX@FG groups after 24 h respectively, indicating the DOX@FG + NIR irradiation increased the intracellular DOX accumulation. Additionally, the mild photothermal effect mediated by NIR laser irradiation also promoted the retention of DOX in MCF‐7/ADR cells (Figure 5d). The retention of DOX in DOX@FG + NIR was 1.52‐ and 1.95‐fold greater than that of DOX@FG after 18 and 24 h respectively, suggesting the FG boosted the retention of DOX in the presence of NIR. Based on the results above, the mild photothermal effect promoted intracellular DOX accumulation and retention by reversing MDR, therefore an increase in apoptosis could be expected.

### 3.6 Apoptosis of MCF‐7/ADR with DOX@FG under NIR

Under NIR irradiation, DOX@FG reversed MDR mechanisms by reducing DOX efflux and combating antiapoptotic signals, increasing the sensitivity of MCF‐7/ADR cells to DOX. To further demonstrate the anti‐tumor efficacy in vitro, flow cytometry was used to determine the apoptosis of different groups (Figures 5e and S10). The apoptosis rate of DOX@FG + NIR group (85.9%) was higher than that of DOX@FG group (63.1%), demonstrating the effectiveness of reversing MDR by downregulating antiapoptotic signals upon NIR irradiation. Collectively, MCF‐7/ADR cells incubated with DOX@FG exhibited excellent DOX accumulation and retention abilities, as well as sensitivity to DOX in the presence of NIR, which was attributed to DOX@FG reversal of the MDR from the pump and nonpump resistance mechanisms.

### 3.7 Biodistribution of DOX@FG in vivo

**FIGURE 6:**
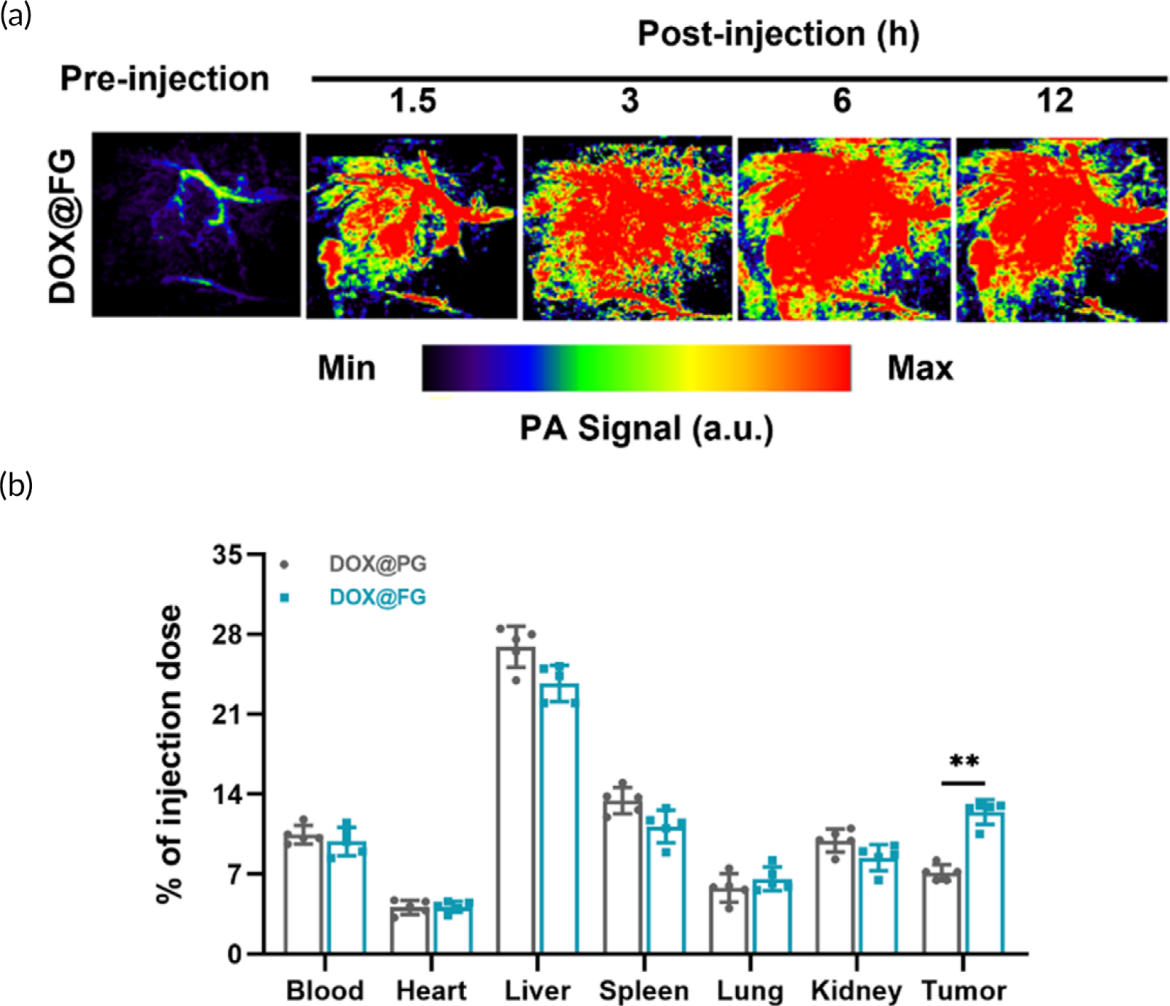
Biodistribution of DOX@FG in major organs and tumor. (a) Photoacoustic tomography images of tumor areas in MCF‐7/ADR tumor‐bearing nude mice that received DOX@FG intravenously. Herein, gold nanorods (GNR) was used as contrast agents. (b) In vivo distribution of the DOX@PG and DOX@FG at 6 h after intravenous injection based on the amounts of Au in various tissues or organs analyzed by ICP‐MS (*n* = 5). **p < 0.001.

To demonstrate the tumor‐targeting potential of DOX@FG, its distribution in MCF‐7/ADR tumor‐bearing nude mice was determined by PA tomography. The tumor site after intravenous DOX@FG administration was imaged in Figure 6a and showed the highest PA intensity signal at 6 h postinjection, indicating the maximum accumulation of DOX@FG in tumor was achieved after 6 h administration. Also, the distribution of DOX@FG and DOX@PG in major organs and tumor were determined after 6 h treatment. As indicated in Figure 6b, the DOX@FG and DOX@PG almost accumulated in the liver and spleen after intravenous injection, which could be attributed to the abundant reticuloendothelial phagocytosis systems in the liver and spleen. Notably, the accumulation of DOX@FG at tumor sites was ~1.75‐fold higher than that of the DOX@PG group. The biodistribution results indicated that DOX@FG could target MCF‐7/ADR cells through FA‐mediated active targeting, thereby enhancing the drug accumulation at tumor sites. Notably, the H&E staining and serum biochemistry showed no significant difference between DOX@FG‐treated mice and healthy mice (Figure S11), indicating that systemic delivery of DOX@FG did not induce noticeable adverse effects.

### 3.8 In vivo antitumor efficacy of DOX@FG

**FIGURE 7:**
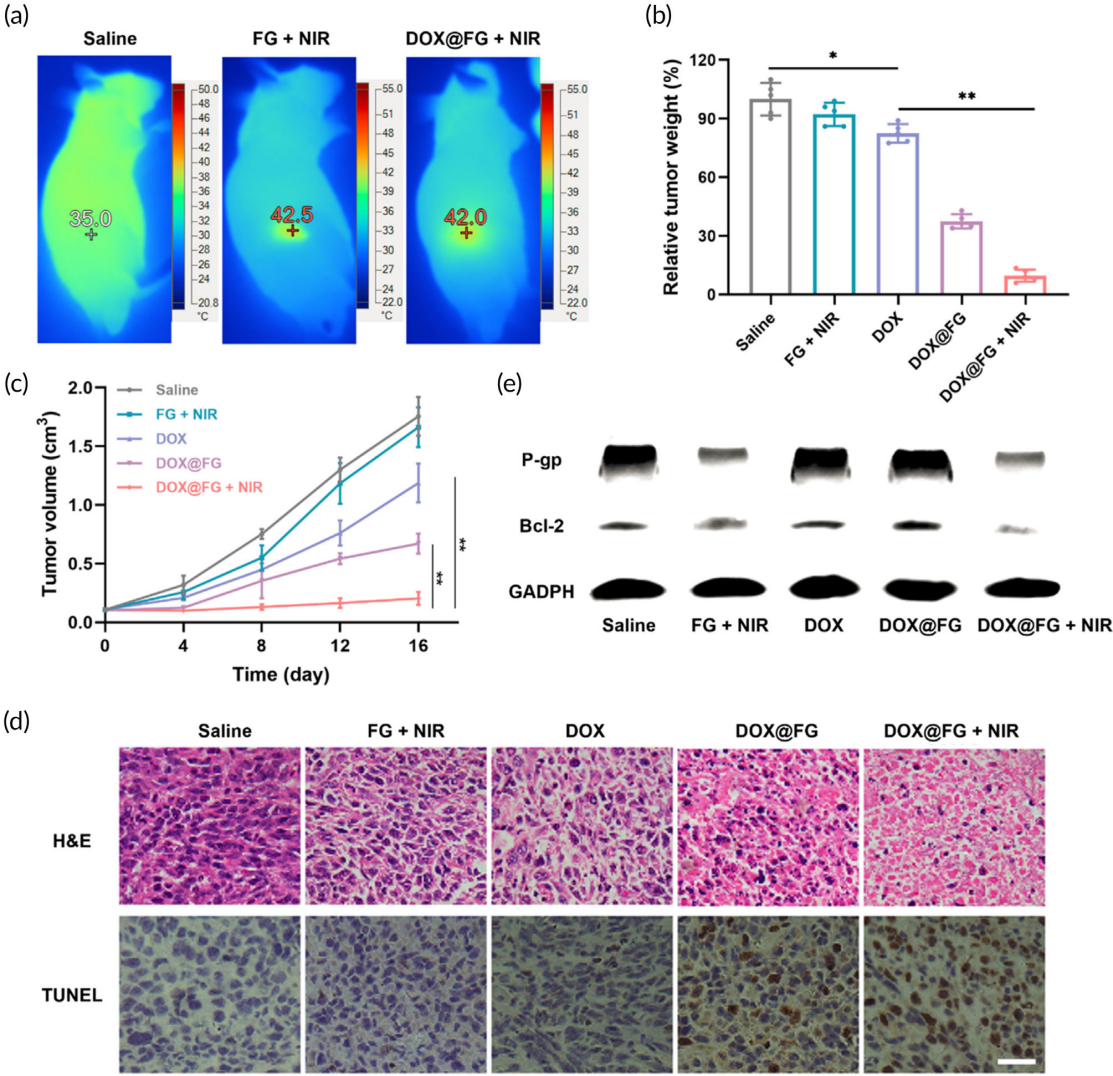
In vivo antitumor efficacy of DOX@FG in BALB/c‐nude mice bearing MCF‐7/ADR cells. (a) The corresponding infrared thermal images of mice receiving saline, FA‐modified GNRs (FG), or DOX@FG. (b) Relative tumor weight measured at the end of the in vivo experiment in the indicated treatments (*n* = 5). (c) Tumor growth profiles of the mice treated with saline, FG + NIR, DOX, DOX@FG, and DOX@FG + NIR (*n* = 6). (d) Images of H&E staining and TUNEL immunohistochemistry of MCF‐7/ADR tumor sections after different treatments, scale bar: 100 μm. (e) Western blotting analysis of the P‐gp and Bcl‐2 levels in MCF‐7/ADR cells under different treatments. *p < 0.05 and **p < 0.001.

The in vivo antitumor efficacy of DOX@FG was evaluated on MCF‐7/ADR tumor‐bearing BALB/c‐nude female mice. After mice were injected with saline, FG, or DOX@FG, the tumors were irradiated with a 808 nm laser at 2.0 W/cm^2^ for 3 min; and corresponding infrared thermal images were obtained. The localized temperature at the tumor site was elevated to 42.0°C and 42.5°C for the DOX@FG and FG groups, respectively, producing a mild photothermal effect (Figure 7a). The antitumor efficacy of DOX@FG integrating chemotherapeutic and mild photothermal effects was further investigated, with saline, DOX and FG as control groups. After treatment with different treatments, the mild photothermal effect induced by FG hardly significantly inhibited tumor growth. Compared to free DOX, DOX@FG inhibited tumor growth in the absence of NIR irradiation because the targeting effect of FA enhanced intracellular DOX accumulation. Notably, under NIR irradiation, the MCF‐7/ADR cells were sensitive to DOX@FG and the tumor weight was 0.25‐fold greater than DOX@FG without NIR (Figure 7b), which attributed to DOX@FG reversing the MDR under mild photothermal treatment. These results were consistent with the tumor growth profiles in Figure 7c. Furthermore, the images of H&E staining and TUNEL immunohistochemistry of MCF‐7/ADR tumor sections demonstrated the excellent anti‐tumor efficacy of DOX@FG under NIR irradiation (Figure 7d). The nuclei of cells in the FG + NIR group were intact, large, and deeply stained, indicating that the mild photothermal process did not cause tumor cell necrosis. Compared with DOX, the DOX@FG + NIR group showed obvious cell necrosis, almost all tumor cells lost their membrane integrity, and only sporadic nuclear fragments were seen. TUNEL immunohistochemistry detected the apoptosis of tumor cells, and the positive TUNEL was brownish yellow. There was almost no brown color in the saline group and FG + NIR group. However, around 50% of the cells in DOX@FG group were TUNEL‐positive. In particular, when combined with NIR irradiation, more than 80% of the TUNEL‐positive cells were observed in DOX@FG + NIR group, suggesting that a large number of drug‐resistant tumor cells underwent apoptosis. These results confirming that the DOX@FG + NIR can improve the sensitivity of drug‐resistant cells to DOX.

To verify the response of MCF‐7/ADR cells to DOX@FG under NIR, the expressions of P‐gp and Bcl‐2 were analyzed by western blotting. Upon NIR irradiation, the levels of P‐gp and Bcl‐2 were downregulated in tumors under DOX@FG treatment (Figures 7e and S12), indicating mild photothermal effect induced by DOX@FG contributed to inhibiting DOX efflux and combating the antiapoptotic pathway. Collectively, the FA‐mediated tumor targeting and GNRs‐induced mild photothermal effect synergistically enhanced DOX accumulation and retention in the MCF‐7/ADR cells, meanwhile sensitizing the MCF‐7/ADR cells to chemotherapy by overcoming MDR in terms of the pump and nonpump resistance mechanisms. The above results indicate that, NIR light can act as an external switch to control the mild photothermal effect for reversing chemotherapy resistance. Notably, the plasmon resonance of GNRs can be tuned from the visible to NIR regions that depend on the nanorod's aspect ratio. The NIR laser irradiation‐based cancer therapy is especially attractive, because GNRs can be easily made to maximally absorb in the “water window,” which shows minimal absorbance by skin and tissue, and thus providing deep tissue penetration with high spatial precision. In the future, nanorod's aspect ratio, irradiation time and power need to be optimized to achieve effective treatment of deep‐seated tumors.

## 4 CONCLUSION

In summary, we constructed a pH and NIR‐responsive nanomedicine DOX@FG by anchoring FA targeting ligands and hydrazone‐cleavable DOX derivatives onto GNRs, effectively inhibiting pump and nonpump mechanisms of MDR to enhance chemotherapy efficacy. DOX@FG with FA modifications enhanced the system's tumor‐targeting ability by recognizing the FR broadly on the MCF‐7/ADR cell surface, followed by pH‐sensitive dissociation to promote DOX release and thus impair cell viability. Moreover, the mild photothermal treatment induced by DOX@FG simultaneously downregulated the level of P‐gp and Bcl‐2, blocking DOX efflux and sensitizing the MCF‐7/ADR cell to DOX. In vivo anti‐tumor results also demonstrated that DOX@FG in the presence of NIR reversed the MDR from the pump and nonpump resistance mechanisms, enhancing the MCF‐7/ADR cell sensitivity and inhibiting the cancer cell growth. Therefore, the DOX@FG is a promising nanomedicine for two‐pronged reversal of chemotherapy resistance to amplify the efficacy of chemotherapy.

## AUTHOR CONTRIBUTIONS

**Qi Shang:** Conceptualization; data curation; formal analysis; methodology; writing – original draft; writing – review and editing. **Ziyan Chen:** Conceptualization; formal analysis; methodology; visualization; writing – original draft; writing – review and editing. **Jing Li:** Conceptualization; formal analysis; methodology; visualization; writing – original draft; writing – review and editing. **Mingmei Guo:** Investigation; writing – original draft; writing – review and editing. **Jiapei Yang:** Investigation; writing – original draft; writing – review and editing. **Zhu Jin:** Investigation; writing – review and editing. **Yuanyuan Shen:** Supervision; writing – review and editing. **Shengrong Guo:** Conceptualization; supervision; writing – original draft; writing – review and editing. **Feihu Wang:** Conceptualization; funding acquisition; project administration; supervision; writing – original draft; writing – review and editing.

## CONFLICT OF INTEREST STATEMENT

The authors declare no competing financial interest.

### PEER REVIEW

The peer review history for this article is available at https://www.webofscience.com/api/gateway/wos/peer-review/10.1002/btm2.10670.

## Supporting information


**Data S1.** Supporting Information.

## Data Availability

The data that support the findings of this study are available from the corresponding author upon reasonable request.
