# Supplementary material for: Two‐pronged reversal of chemotherapy resistance by gold nanorods induced mild photothermal effect
Source: Bioeng Transl Med. 2024 Apr 18;9(5):e10670. doi: 10.1002/btm2.10670 (PMC11561791; doi:10.1002/btm2.10670)
Supplement: Supplementary file 1 — Data S1. Supporting Information. [file BTM2-9-e10670-s001.docx]

Supporting Information for

**Two-pronged reversal of chemotherapy resistance by gold nanorods induced mild photothermal effect**

Qi Shang^1^^, #^, Ziyan Chen^2, #^, Jing Li^3, #^, Mingmei Guo^1^, Jiapei Yang^1^, Zhu Jin^2^, Yuanyuan Shen^2^, Shengrong Guo^2*^, Feihu Wang^1,2*^

^1^School of Biomedical Engineering, Shanghai Jiao Tong University, 800 Dongchuan Road, Shanghai 200240, PR China.

^2^School of Pharmacy, Shanghai Jiao Tong University, 800 Dongchuan Road, Shanghai 200240, PR China.

^3^Department of Pharmacy, Putuo People’s Hospital, 1219 Jiangning Road, Shanghai 200060, PR China.

^#^These authors contributed equally to this work.

*Corresponding author. E-mail: srguo@sjtu.edu.cn (Shengrong Guo); fhwang21@sjtu.edu.cn (Feihu Wang)


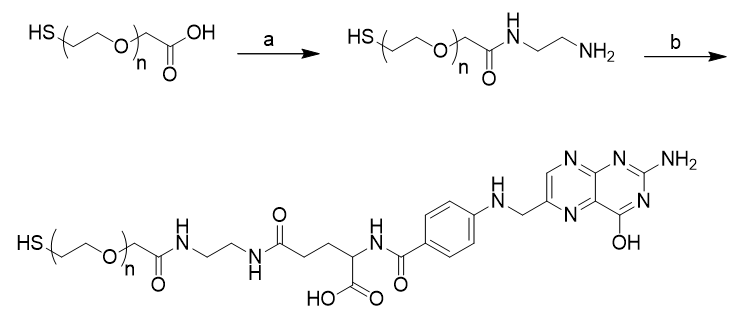


**FIGURE S1** Synthesis scheme of FA-PEG-SH. a) Ethylenediamine, EDC, NHS; b) Folic acid, EDC, NHS.


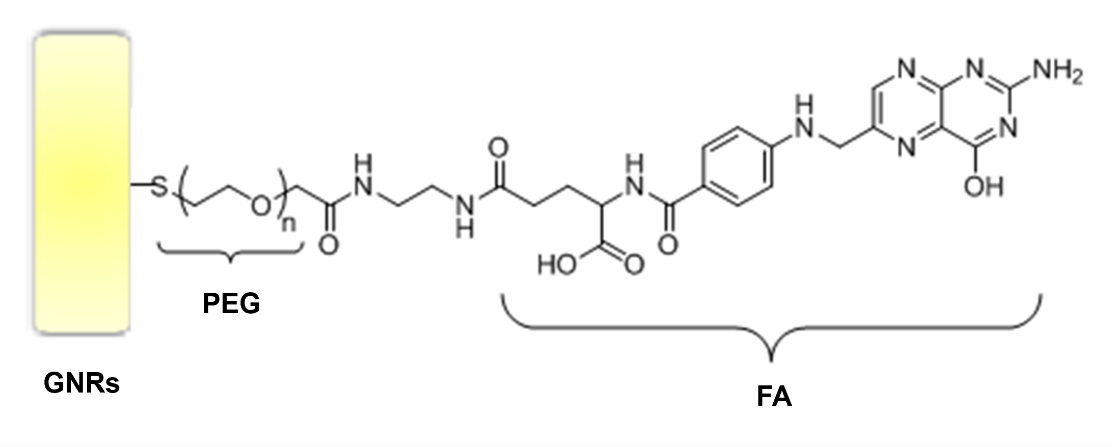


**FIGURE S2** Detailed chemical structure of FG (FA-PEG-GNRs).


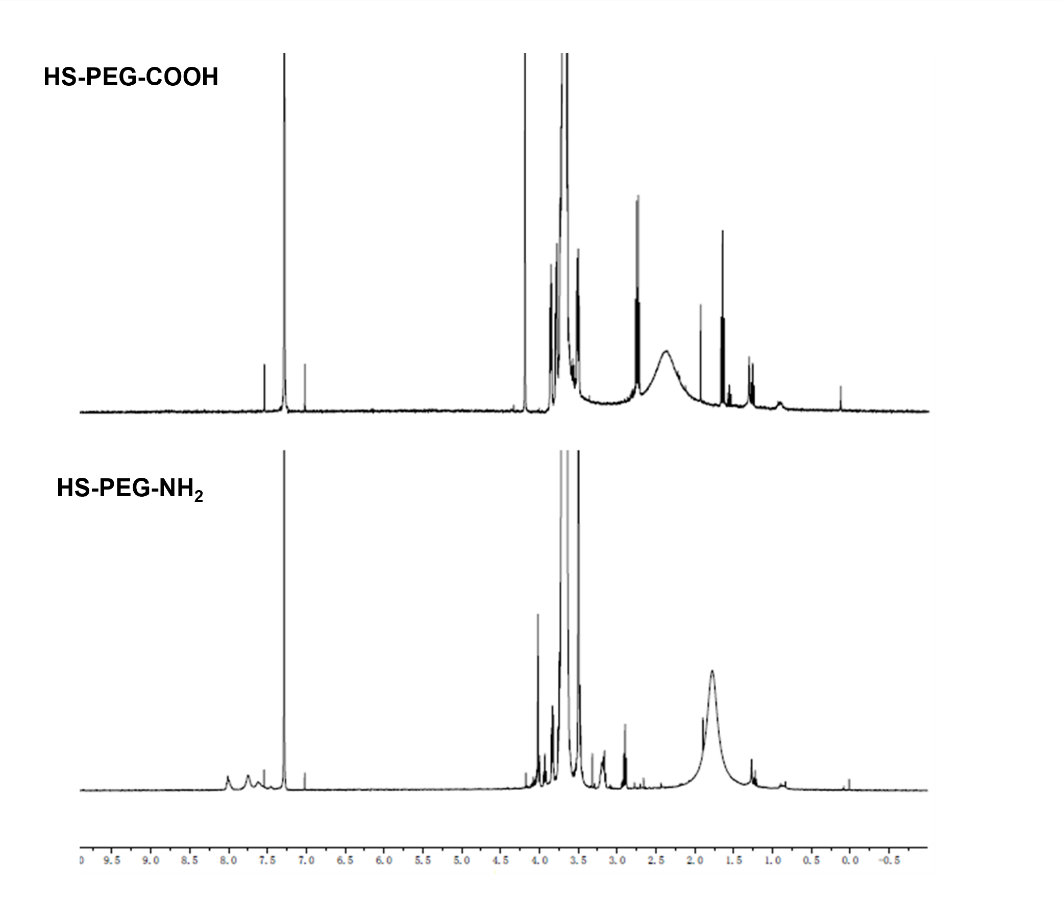


**FIGURE S3** ^1^H NMR spectrum of HS-PEG-COOH and HS-PEG-NH_2._


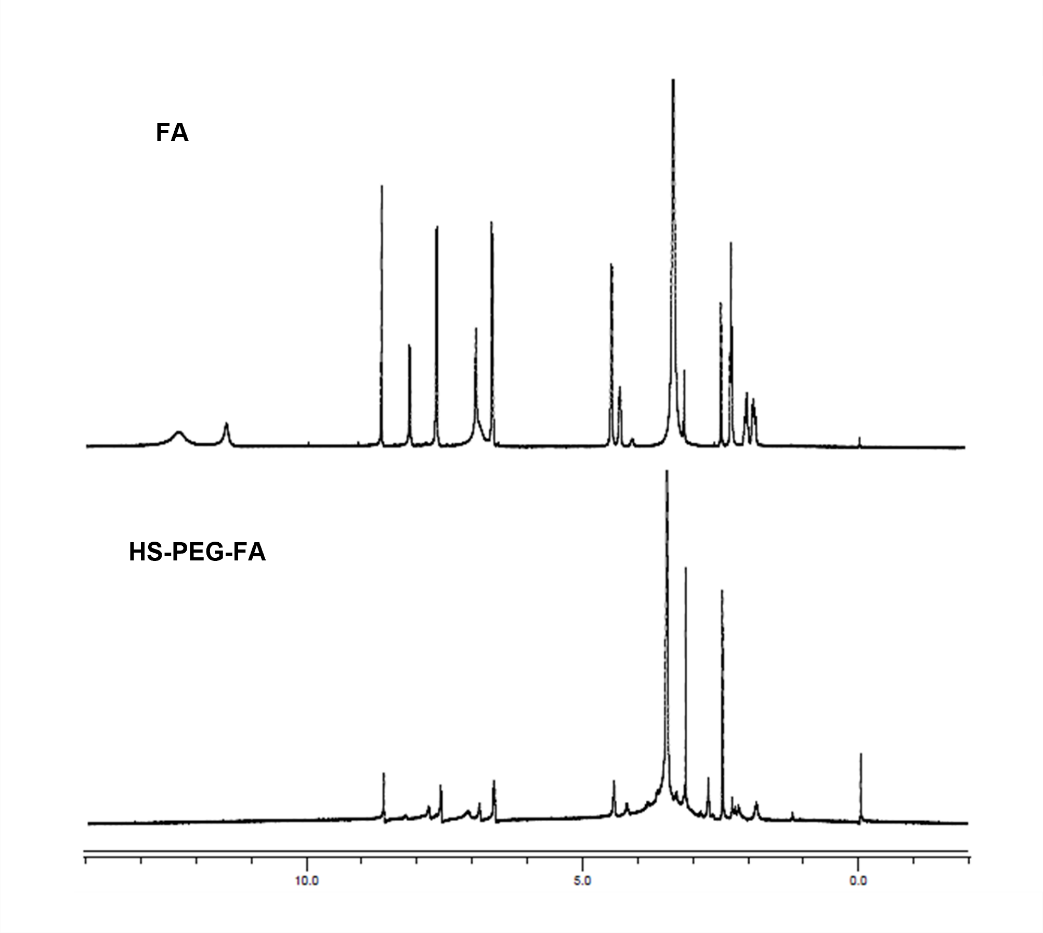


**FIGURE S4** ^1^H NMR spectrum of FA and HS-PEG-FA.


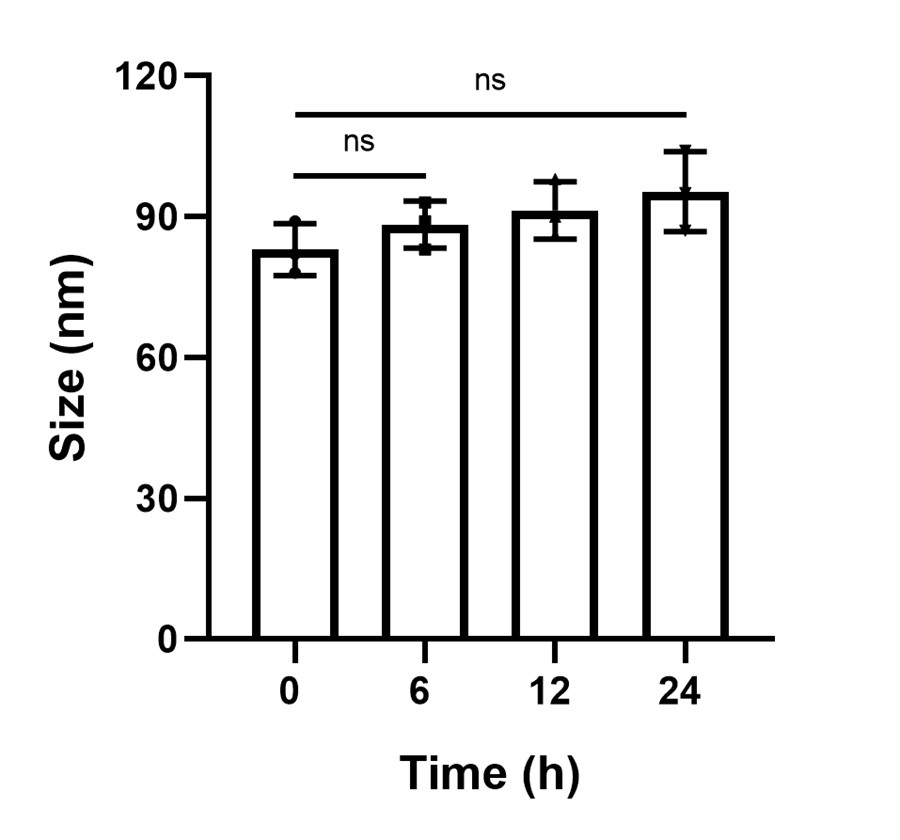


**FIGURE S5** The particle size of DOX@FG incubated in PBS with 10% FBS at 37℃ for different periods of time (n = 3), ns means no significant difference.

**
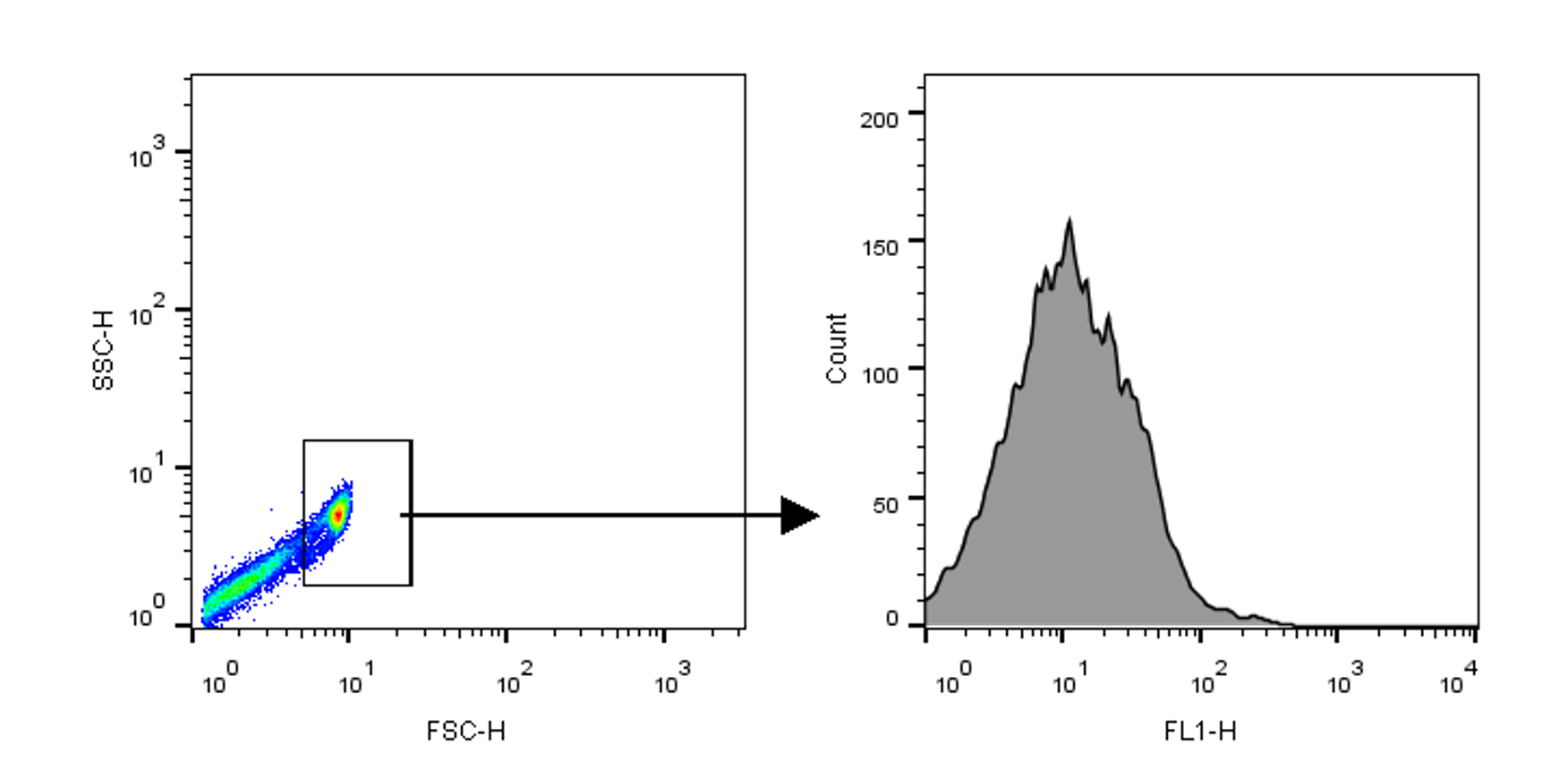
**

**FIGURE S6.** Gating strategy for flow cytometric analysis of DOX mean fluorescence intensity in MCF-7/ADR cells. The events were gated on FSC-H vs. SSC-H to exclude cell debris, the histogram showed the fluorescence signal at FL1-H channel on FSC-H/SSC-H gated events.


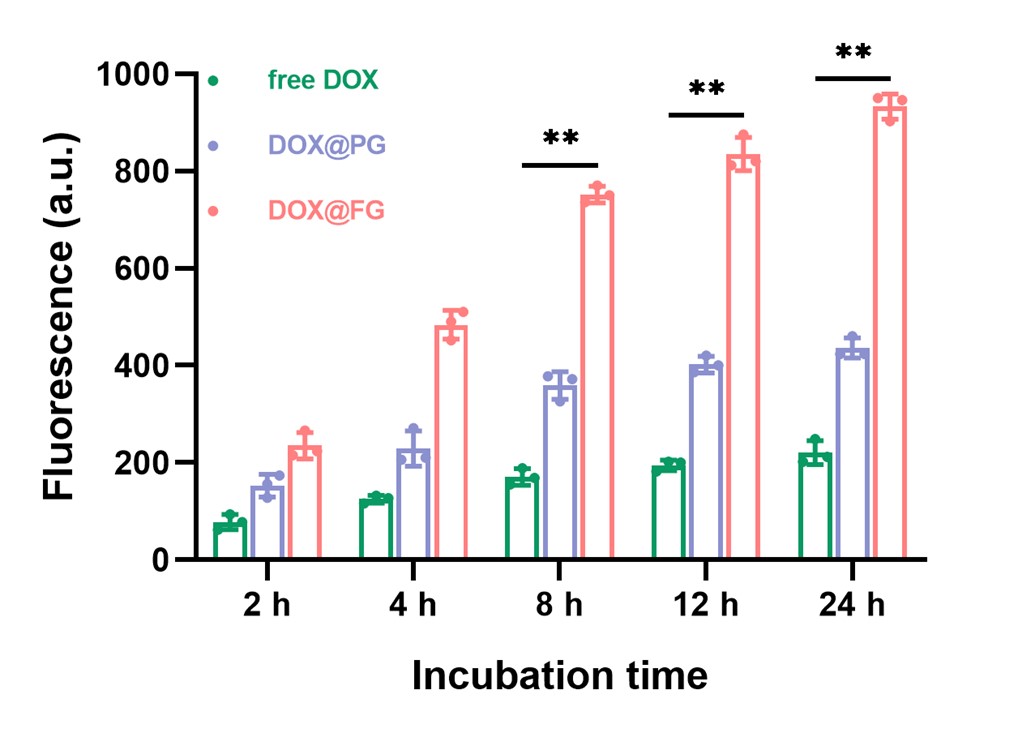


**FIGURE S7** Flow cytometry determined mean fluorescence intensity of MCF-7 cells after incubation with free DOX, DOX@PG or DOX@FG for various timepoints (n = 3). The dose of DOX was 2.5 μg/mL. **p < 0.001.


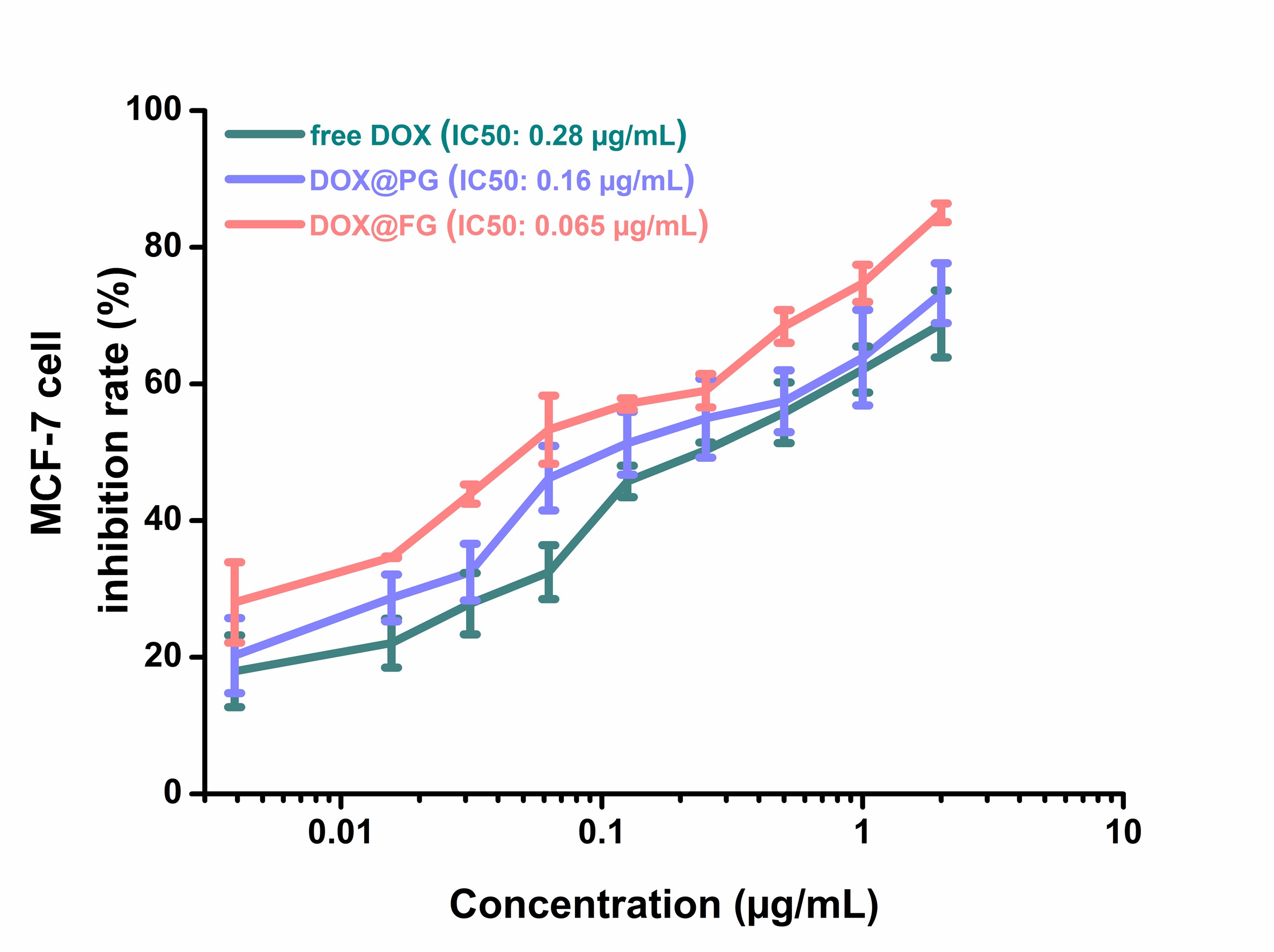


**FIGURE S8** The inhibition rate of MCF-7 cells incubated with free DOX, DOX@PG or DOX@FG after 48 h (n = 3).


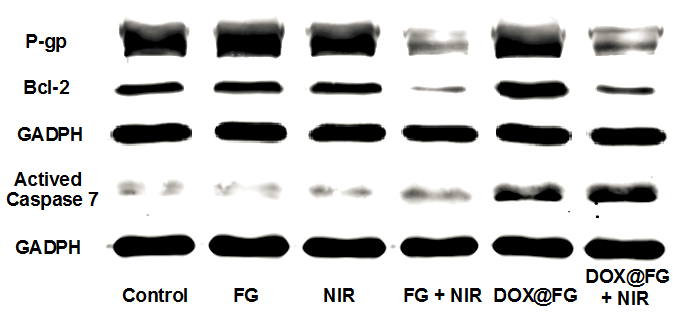


**FIGURE S9** The original western blotting image of the P-gp, Bcl-2, and Caspase 7 in MCF-7/ADR cells upon different treatments.


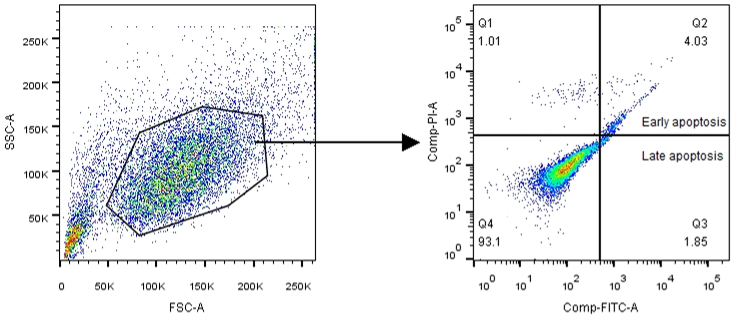


**FIGURE S10** Gating strategy for flow cytometric analysis of apoptosis of MCF-7/ADR cells. The events were gated on FSC-H vs. SSC-H to exclude cell debris, the two-parameter density plot showed the gated events stained with FITC and PI to evaluate cell apoptosis on Comp-FITC-A vs. Comp-PI-A.


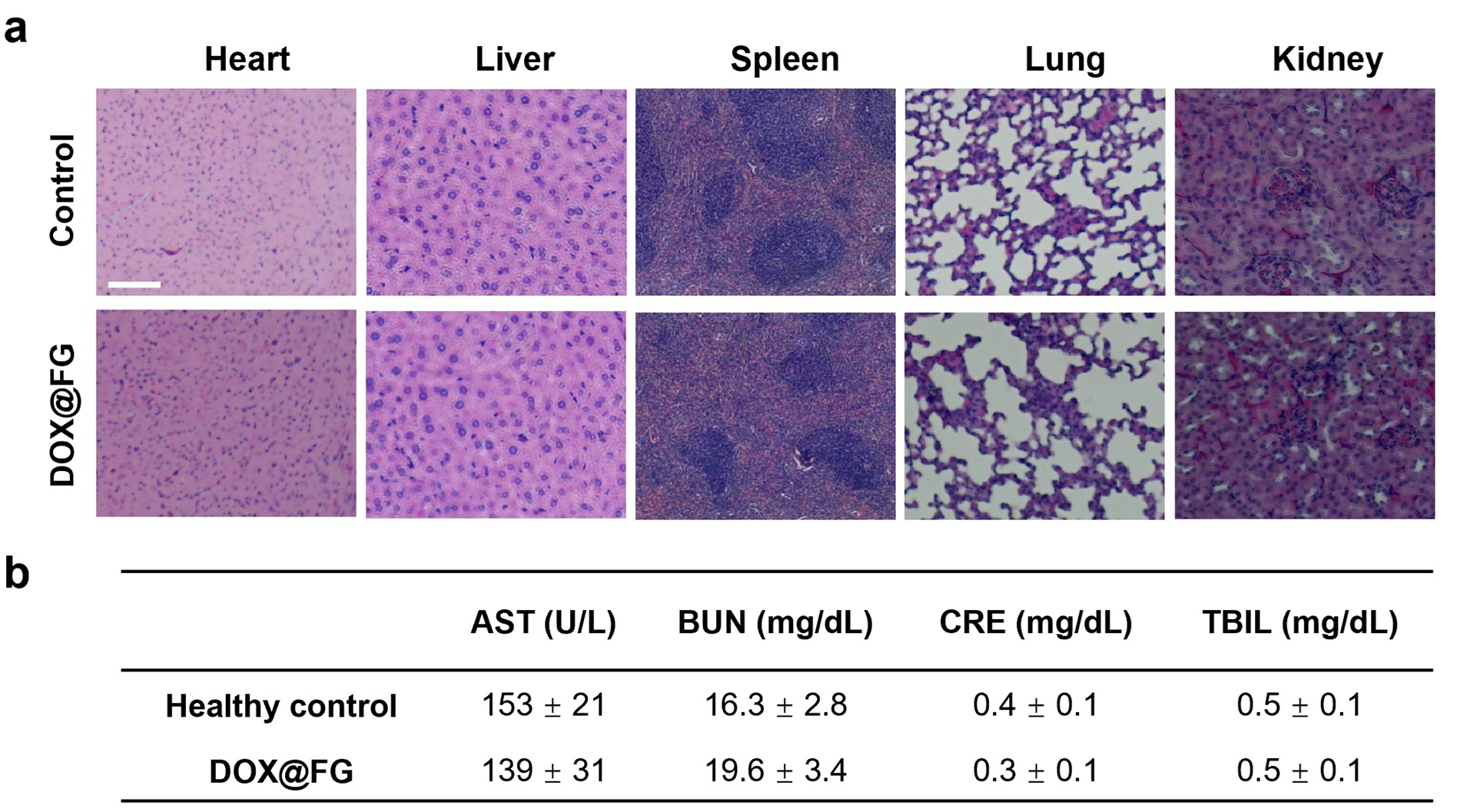


**FIGURE S11** Safety studies of DOX@FG treated mice. (a) H&E staining of major organs collected from healthy mice and DOX@FG treated mice, scale bar: 200 µm. (b) Serum biochemistry of aspartate aminotransferase (AST), blood urea nitrogen (BUN), creatinine (CRE), and total bilirubin (TBIL) in healthy mice and mice treated with DOX@FG treatment. Data were presented as mean ± SD (n = 3).


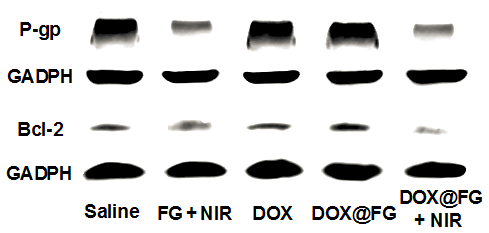


**FIGURE S12** The original western blotting image of the P-gp and Bcl-2 levels in MCF-7/ADR tumors after the mice were treated with saline, FG, DOX and DOX@FG in the presence or absence of NIR laser irradiation.
